# Unveiling the Fragrant Secrets of *Dendrobium devonianum*: Terpenoid Pathways and Floral Scent Dynamics

**DOI:** 10.3390/metabo16040276

**Published:** 2026-04-18

**Authors:** Shichao Wang, Shu He, Congjun Yuan, Xingliang Chen, Hoang Van Sam, Wei Chen Lum, Yaquan Dou, Rui Shi

**Affiliations:** 1 Yunnan Provincial Key Laboratory for Conservation and Utilization of In-Forest Resources, Kunming 650224, Chinashirui@swfu.edu.cn (R.S.); 2 International Joint Laboratory of Southeast Asian, Southwest Forestry University, Kunming 650224, China; 3 Guizhou Academy of Forestry, Guiyang 550005, China; 4 Research Institute of Forest Policy and Information, Chinese Academy of Forestry, Beijing 100010, China; 5 Forest Plant Department, Vietnam National University of Forestry, Xuan Mai, Ha Noi 13400, Vietnam; 6 Department of Bio and Natural Resource Technology, Faculty of Bioengineering and Technology, Universiti Malaysia Kelantan, Kota Bharu 17600, Kelantan, Malaysia; 7 Yunnan Gaoligong Green Industry Innovation Institute, Tengchong 679100, China

**Keywords:** *Dendrobium devonianum*, aromatic compounds, volatile organic compounds, volatile esters, terpenoids

## Abstract

Background/Objectives: The orchid *Dendrobium devonianum* Paxt., valued for its ornamental and medicinal properties, is widely used in horticulture, medicine, and food industries. Methods: This study investigated dynamic changes in aroma-active volatile organic compounds (VOCs) and associated gene expression in *D. devonianum* flowers across four developmental stages (bud, half bloom, full bloom, and aging) using headspace solid-phase microextraction, gas chromatography–mass spectrometry, and transcriptome analysis. Results: Floral VOCs, particularly volatile terpenoids and esters, were most abundant at full bloom. Among the 664 VOCs identified, α-hemelene, β-bisabolene, δ-naphthalene, perillyl alcohol, L-perillyl alcohol, terpinen-4-ol, 2-(4-methylphenyl)propan-2-ol, cis-3-hexenyl butyrate, and α-pinene were likely to contribute to floral scent. Terpene biosynthesis pathways played a pivotal role in floral fragrance formation. A comprehensive terpenoid biosynthesis pathway for *D. devonianum* floral scent was proposed, and eight genes encoding key regulatory enzymes were identified. Conclusions: These results provide new insights into terpenoid metabolism in Dendrobium and may guide future research on the utilization of floral scent.

## 1. Introduction

Dendrobium, family of Orchidaceae, is widely used as an ornamental plant for decorating gardens, homes and other environments, and as a medicinal plant for managing hyperglycemia and improving immunity [1,2]. *Dendrobium devonianum* Paxt., distributed in southwestern China [3], has been employed for its potential in medicine and food, including treatment of gastrointestinal disorders [4,5,6,7]. The flowers of *D. devonianum* are used in traditional soups and teas [8,9]. They are rich in amino acids, fatty acids and flavonoids, such as the volatile fatty acid ester methyl linoleate and the flavonoid quercetin [10]. These findings suggest that *D. devonianum* flowers contain bioactive compounds of potential interest for further research into functional foods and medicinal applications, pending comprehensive sensory evaluation and safety assessment.

The flowers of *D. devonianum* exhibit a distinct floral scent, an important characteristic of higher plant floral organs that strongly influences consumer preference [11]. Plant volatile organic compounds (VOCs) play key roles in attracting pollinators and defending against biotic and abiotic stress [12]. Common VOCs include terpenes, phenylpropanoids and fatty acid derivatives, the latter mainly consisting of small molecules such as alcohols, aldehydes and lipids [12]. VOC composition and concentration are highly dependent on environmental conditions and the developmental stage of plant tissues, showing spatiotemporal specificity [13]. Chen et al. reported that characteristic scent compounds in Rosa x hybrida cv. ‘Yves Piaget’ peak at full bloom [14]. Similarly, in *Rosa canina* L., terpenoid abundance peaks at the newly opened stage, accompanied by increased expression of corresponding genes [15]. Another study indicated that white flowers of *R. canina* L. exhibit higher abundances of floral scent compounds than deep pink flowers; white petals also contain higher levels of total flavonoids and phenolics but lower anthocyanin content, suggesting a potential association between compounds influencing flower color and those responsible for floral scent [16].

A number of studies have investigated the scent of Dendrobium flowers. Li et al. analyzed volatile terpenoids in two *Dendrobium officinale* flowers with distinct aromas [17]; Du et al. explored the molecular mechanisms underlying floral scent formation in *Dendrobium chrysotoxum* [18]; moreover, Yang et al. characterized the volatile components of *Dendrobium moniliforme* (L.) Sw. and *Dendrobium nobile* “H1” [19]. These studies consistently showed that terpenoids play an important role in the formation of Dendrobium floral aroma. Terpenoids also serve as key ingredients in the flavor and fragrance industry [20]. Despite these studies, the key aroma-active compounds and their underlying biosynthetic mechanisms remain insufficiently characterized. This knowledge gap limits our understanding of the metabolic regulation of floral scent and constrains further exploration of the potential applications of *D. devonianum* flowers.

In this study, we analyzed the VOCs in *D. devonianum* flowers at four distinct floral growth stages (bud, half-bloom, full bloom, and aging). By combining VOC profiling with transcriptomic data, we identified the principal constituents and key compounds contributing to floral scent and explored the molecular mechanisms underlying their formation. This work aimed to provide a comprehensive understanding of the metabolic regulation of floral aroma in *D. devonianum* and to inform potential applications of its VOCs, including in food-related industries.

## 2. Materials and Methods

### 2.1. Plant Materials

*D. devonianum* flowers were obtained from the Dendrobium Germplasm Resource Conservation Research Center, Longling County, Baoshan City, Yunnan Province, China (24°6297′ N, 98°6742′ E, 1480 m a.s.l.). The specimens were identified as *D. devonianum* (Orchidaceae) by qualified botanists from the Botany Teaching and Research Section of Southwest Forestry University.

**Figure 1:**
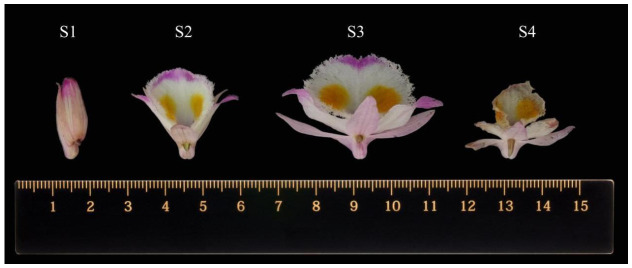
Four developmental stages of *Dendrobium devonianum* flowers. S1: bud stage; S2: half-bloom stage; S3: full-bloom stage; S4: aging stage.

Flowers were collected randomly at four developmental stages: bud (S1), half-bloom (S2), bloom (S3), and aging (S4) (Figure 1). All flowers were healthy and undamaged. For each developmental stage, three independent biological replicates were prepared, with each replicate consisting of flowers collected from different individual plants. Samples were immediately frozen in liquid nitrogen and stored at −80 °C until further analysis.

### 2.2. Sample Preparation and Gas Chromatography–Mass Spectrometry (GC–MS) Analysis

For solid-phase microextraction (SPME), flower samples, previously stored in liquid nitrogen, were retrieved and ground into a fine powder under liquid nitrogen, followed by thorough mixing via vortex. An aliquot of 0.5 g of the powder was accurately weighed and placed into a 25 mL headspace vial. Saturated sodium chloride solution and 10 mL of internal standard solution were subsequently added. The vial was then transferred to an automated headspace sampler for GC-MS analysis, employing spitless injection mode. The extraction procedure was conducted under constant temperature at 60 °C with agitation for 5 min. A 120 µm DVB/CWR/PDMS fiber (Supelco, Bellefonte, PA, USA) was inserted into the headspace vial for extraction over 15 min, followed by desorption at 250 °C for 5 min [21].

VOCs were analyzed using an Agilent 7890B gas chromatograph coupled with a 7000D mass spectrometer (Agilent Technologies, Santa Clara, CA, USA), equipped with a 30 m × 0.25 mm × 1.0 μm DB-5 MS capillary column (5% phenyl polymethylsiloxane, Agilent Technologies). Helium was used as the carrier gas at a linear velocity of 1.0 mL/min. The oven temperature was initially held at 40 °C for 3.5 min, then ramped at 6 °C/min to 280 °C and held for 5 min. The injector and detector temperatures were 250 °C and 280 °C, respectively. Mass spectrometry was performed in electron impact (EI) mode at 70 eV, scanning m/z 30–350 at 1 s intervals, with the quadrupole, ion source, and transfer line maintained at 150 °C, 230 °C, and 280 °C, respectively. VOCs were putatively identified by comparing mass spectra with the National Institute of Standards and Technology (NIST) library and by calculating linear retention indices.

### 2.3. Differential Aroma-Active VOCs

The relative abundances of VOCs were first standardized using unit variance scaling. Multivariate statistical analyses were then performed to evaluate differences among flower developmental stages. Principal component analysis (PCA) was applied to visualize the overall relationships among samples, while partial least-squares discriminant analysis (PLS-DA) was employed to highlight VOCs that contributed significantly to the differences between stages. Differential aroma-active VOCs were identified based on a variable importance in projection (VIP) score ≥ 1 and |Log2(fold change)| ≥ 1.

The odor activity value (OAV) of each VOC was calculated by dividing its relative abundance by its odor threshold in water [22], with OAV ≥ 1 considered indicative of a potent odorant. The differential aroma-active VOCs were annotated for their odor characteristics by cross-referencing the published literature and online databases, including the Good Scents Company (http://www.thegoodscentscompany.com; accessed on 29 January 2025), Flavornet (http://www.flavornet.org; accessed on 29 January 2025), and the Food Flavor Laboratory (http://foodflavorlab.cn/#/home; accessed on 29 January 2025).

### 2.4. RNA Extraction, Sequencing (RNA-Seq) and Transcriptome Analysis

Total RNA was extracted from *D. devonianum* flowers using a plant RNA isolation kit (Thermo Fisher Scientific, Waltham, MA, USA) following the manufacturer’s instructions. First- and second-strand cDNA synthesis was performed using random primers, SuperScript II reverse transcriptase, DNA polymerase I, and RNase H according to the manufacturer’s protocols. Three biological replicates were collected for each developmental stage (S1–S4), and twelve cDNA libraries in total were constructed and sequenced on an Illumina HiSeq platform (HiSeq 2500, Illumina, San Diego, CA, USA).

Raw sequencing reads were filtered to remove adaptor sequences, poly-N reads, and low-quality reads. The resulting clean reads were assembled de novo into transcripts using Trinity, and redundant sequences were consolidated into unigenes. Due to the lack of publicly available reference genome data for *D. devonianum*, a de novo transcriptome assembly strategy was employed. The quality of the sequencing data was assessed by calculating Q30 scores and GC content. Functional annotation of unigenes was performed using Gene Ontology (GO) analysis, while Kyoto Encyclopedia of Genes and Genomes (KEGG) pathway annotation was conducted using Blastball (v2.2.26). Differentially expressed genes (DEGs) were identified at thresholds of FDR < 0.001 and FC > 2. For GO and KEGG pathway analyses, significance was evaluated at a level of *p* < 0.05.

### 2.5. Quantitative Reverse-Transcription Polymerase Chain Reaction (qRT-PCR) Analysis

To validate the RNA-Seq results, eleven genes related to metabolite biosynthesis in flowers were selected for qRT-PCR analysis. These genes were differentially expressed (|log2(Fold Change)| ≥ 1, false discovery rate (FDR) < 0.05), and annotated in sesquiterpenoid and triterpenoid biosynthesis, as well as in limonene and pinene degradation pathways. They were chosen to represent diverse expression patterns, including upregulated, downregulated, and mid-stage peak expression, ensuring comprehensive validation of transcriptional dynamics during floral development. cDNA input was normalized using GAPDH as an internal reference. Gene-specific primers were designed with Primer 3.0, and the primer sequences are listed in Table S1. qRT-PCR was performed using ChamQ Universal SYBR qPCR Master Mix (Vazyme, Nanjing, China) following the manufacturer’s instructions. Standard curves were generated from 10-fold serial dilutions of cDNA. All reactions were performed in three biological replicates, each with three technical replicates. Relative gene expression levels were calculated using the 2^−ΔΔCt^ method. RNA-Seq-derived FPKM values were log2-transformed.

### 2.6. Statistical Analysis

All experiments, including GC-MS, RNA-Seq, and qRT-PCR, were performed with three biological replicates. Student’s *t*-test was used for pairwise comparisons, and differences were considered statistically significant at *p* < 0.05. Statistical analyses were conducted using GraphPad Prism 5, Excel 2013, and SPSS 20.0.

## 3. Results

### 3.1. Volatile Organic Compounds in D. devonianum Flowers

**Figure 2:**
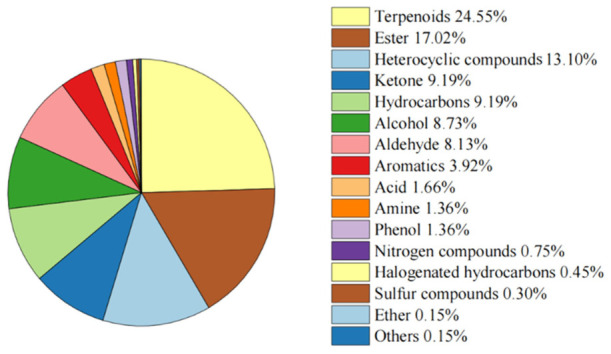
Classification and relative proportions of the 664 volatile organic compounds (VOCs) detected in *D. devonianum* flowers across four developmental stages.

A total of 664 VOCs were identified across the four stages, including terpenoids, esters, heterocyclic compounds, ketones, hydrocarbons, alcohols, and others. As shown in Figure 2, terpenoids represented the largest proportion of VOCs (24.55%), followed by esters (17.02%) and heterocyclic compounds (13.10%). Most VOCs exhibited sweet or fruity odors. Across all stages, the most abundant compounds were 2-isobutyl-3-ethoxypyrazine and hexanal.

**Figure 3:**
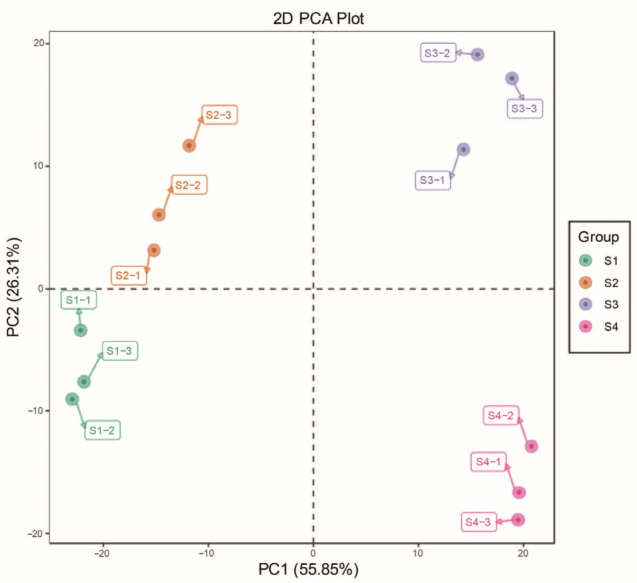
Principal component analysis (PCA) of samples from the four flowering stages based on headspace solid-phase microextraction, gas chromatography–mass spectrometry analysis. Each developmental stage was represented by three biological repeats, denoted as -1, -2, and -3.

**Figure 4:**
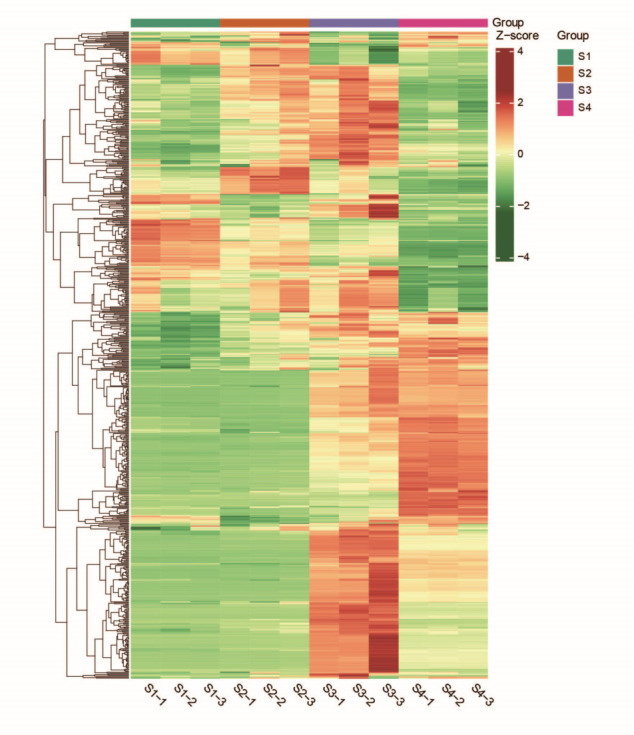
Accumulation pattern of VOCs across developmental stages.

Principal component analysis (PCA) revealed that PC1 accounted for 55.85% of the total variance, while PC2 explained 26.31% and clearly separated the four flowering stages (S1–S4) (Figure 3). These findings indicate stage-dependent variation in VOC composition. Hierarchical clustering analysis of VOCs identified two major clusters: S1 and S2 grouped together, and S3 and S4 formed a separate cluster. Most VOCs showed higher abundance at S3 compared with other stages (Figure 4). Overall, the magnitude of VOC changes increased with floral maturity, suggesting that the blooming stage represents a critical period for VOC accumulation.

### 3.2. Analysis of VOCs at Different Stages of Floral Growth

**Figure 5:**
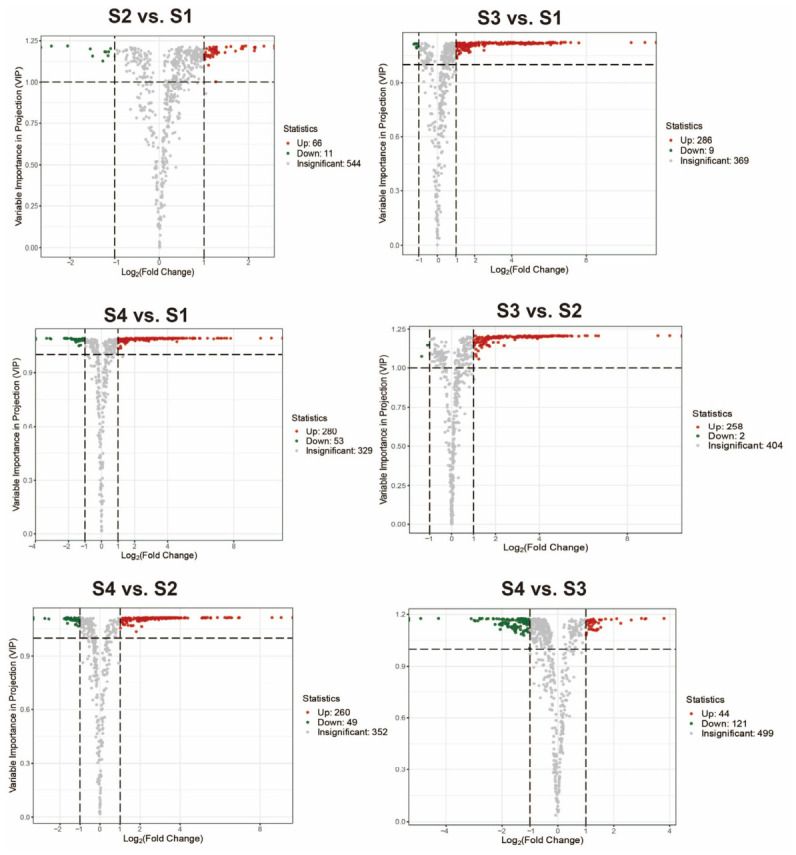
Volcano plots of differential aroma-active VOCs in *D. devonianum*. Green and red dots represent down- and upregulated VOCs, respectively, in each comparison group.

The differential accumulation of VOCs across the four developmental stages of *D. devonianum* was assessed using pairwise comparisons between stages. Most differential aroma-active VOCs were upregulated from S1 to S3 and downregulated from S3 to S4, reflecting their potential contributions to stage-specific floral scent (Figure 5).

S3 had the highest number of upregulated aroma-active VOCs among the four stages. Terpenoids were the major component, accounting for 38.81% in S1–S3 and 33.06% in S3–S4, followed by fatty acid derivatives. Notably, 41 aroma-active VOCs (approximately 20% of the total) absent in S1 peaked in S3, with volatile esters representing the largest proportion. Among these, isobutyric acid, cis-3-hexenyl butyrate, allyl 3-cyclohexylpropionate, and (3Z)-3-hexen-1-yl hexanoate have a strong fruity scent and are edible flavoring compounds. These esters likely contributed most to the floral scent during S3 and represent flavor-active compounds in edible *D. devonianum* flowers.

Based on the threshold of OAV ≥ 1, five VOCs were identified as putative key contributors to the floral scent of *D. devonianum*: perillyl alcohol, L-perillyl alcohol, terpinen-4-ol, 2-(4-methylphenyl)propan-2-ol, and cis-3-hexenyl butyrate. Of these, cis-3-hexenyl butyrate had an OAV of 20, contributing greatly fresh and fruity scents (Table 1). These results showed that terpenoids play a key role in the scent of *D. devonianum* flowers, with the most abundant terpenoid components detected during the blooming stage.

### 3.3. RNA-Seq Analysis of Different Floral Development Stages

The mechanisms underlying the floral scent of *D. devonianum* flowers were further explored by transcriptome analysis. RNA was sequenced from flower samples across the four stages generating a total of 192,664,941 bp of clean data following quality control. Sequencing quality was high, with an overall error rate of 0.03%, Q20 > 95%, Q30 > 90%, and GC content of approximately 44%. De novo assembly of the clean reads yielded 122,331 unigenes with an average length of 1263 bp, an N50 of 1855 bp, and an N90 of 588 bp. Sequences ranging from 200 to 2000 bp accounted for 81.5% of all unigenes. Detailed RNA-seq sequencing and assembly statistics are presented in Tables S2 and S3.

**Figure 6:**
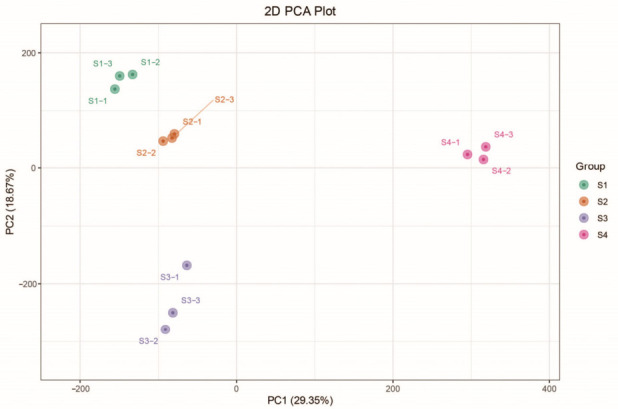
Principal component analysis (PCA) of the transcriptome across four flowering stages (S1–S4). Each stage included three biological replicates, labeled as -1, -2, and -3.

Principal component analysis (PCA) of the transcriptome data showed patterns generally consistent with the metabolome results. Samples from S1–S4 were clearly separated along PC1 and PC2 (Figure 6), indicating distinct gene expression profiles across developmental stages. Notably, the most pronounced changes in gene expression occurred at S3 and S4.

**Figure 7:**
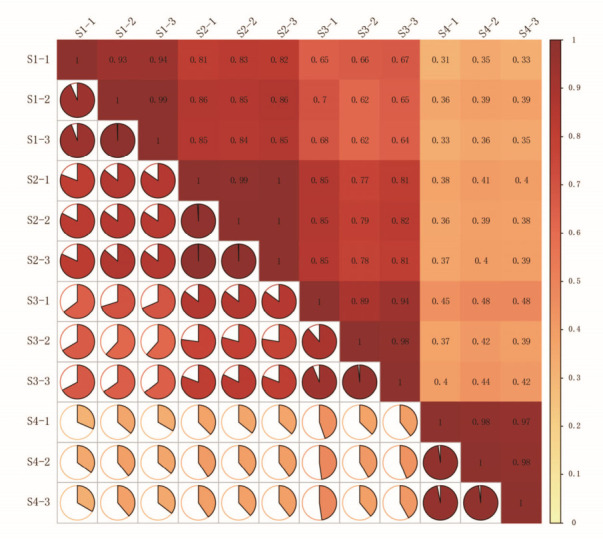
Sample-to-sample correlation analysis among different floral development stages. Numbers in boxes represent correlation coefficients between each pair of samples. Darker colors indicate stronger correlations. Pie charts indicate the proportions of the correlation coefficients. Each flower developmental stage had three biological repeats, denoted as -1, -2 and -3.

As shown in the correlation heatmap (Figure 7), biological replicates within each stage exhibited correlation coefficients (r) greater than 0.85, indicating high reproducibility and reliability of the transcriptome data.

**Figure 8:**
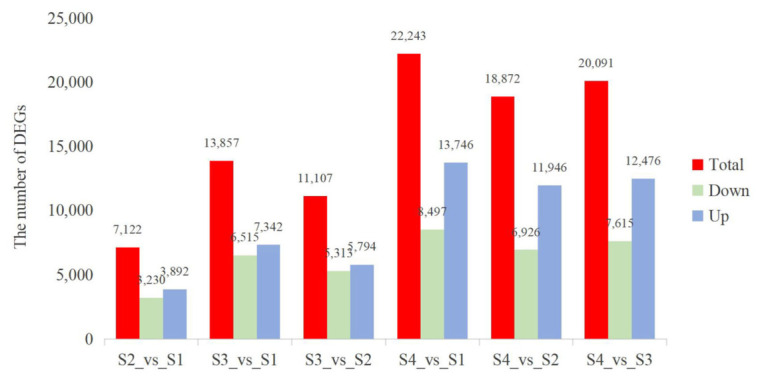
Bar plots showing the number of differentially expressed genes (DEGs) between floral developmental stages.

DEGs from the four samples were compared between groups. As shown in the figure, the total number of DEGs exhibited a clear upward trend from S1 to S4, with the lowest total DEGs (7122) observed in the S2 vs. S1 comparison, followed by 11,107 in S3 vs. S2, 13,857 in S3 vs. S1, 18,872 in S4 vs. S2, 20,091 in S4 vs. S3, and the highest total DEGs (22,243) in the S4 vs. S1 comparison. This progressive increase in DEGs number reflects the enhanced transcriptional differences as the developmental stage advances from S1 to S4. Additionally, across all comparison groups, the number of upregulated DEGs was consistently higher than that of downregulated DEGs: for example, in S2 vs. S1, there were 3892 upregulated and 3230 downregulated DEGs; in S4 vs. S1, the upregulated DEGs reached 13,746, while the downregulated DEGs were 8497 (Figure 8). This consistent pattern indicates that transcriptional activation is the dominant response during the progression from S1 to S4.

**Figure 9:**
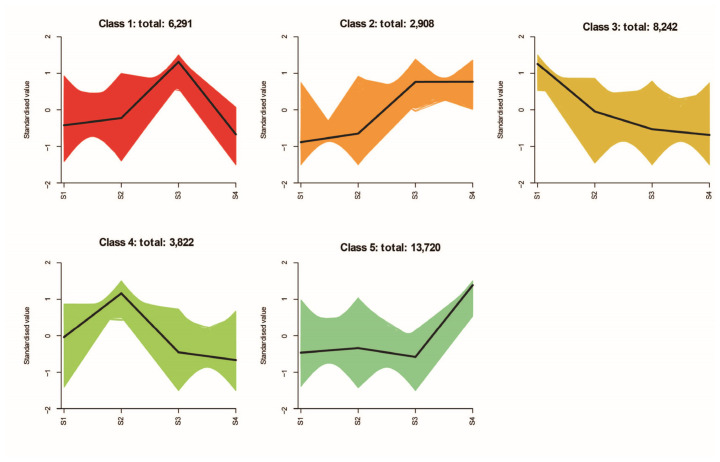
K-means clustering of DEGs across the four floral developmental stages (S1–S4). The horizontal axis represents developmental stages, and the vertical axis shows standardized expression levels. The colored shaded areas represent the fluctuation ranges of the indicators for each cluster, while the black lines indicate the mean trends. Different colors indicate the average expression patterns of DEGs in each cluster, with the number of DEGs in each class represented on the vertical axis.

The expression patterns of DEGs across the floral developmental stages were further analyzed using K-means clustering, resulting in five distinct classes (Figure 9). From S1 to S3, DEGs in Class 1 and Class 2 exhibited an increasing trend. DEGs in Class 1 peaked at S3 and decreased markedly from S3 to S4, whereas those in Class 2 showed a relatively stable pattern from S3 to S4. Notably, the expression pattern of Class 1 mirrored the accumulation trend of VOCs in *D. devonianum* flowers, suggesting that these genes may contribute to scent formation or participate in the associated metabolic pathway.

**Figure 10:**
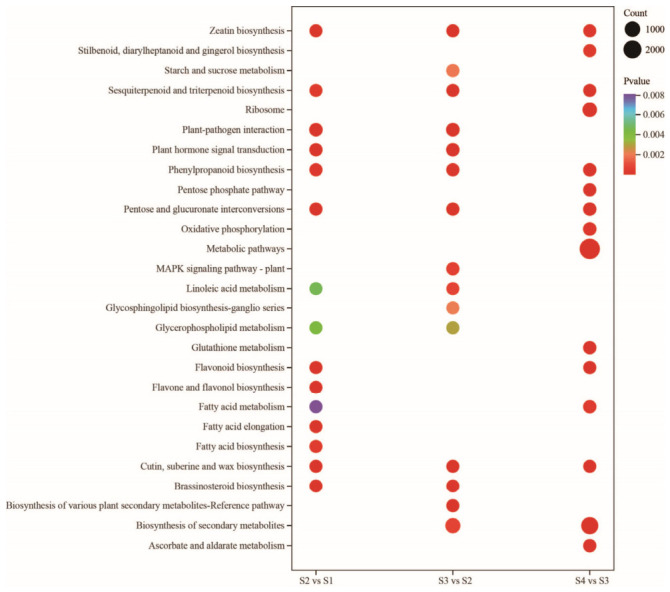
KEGG pathway enrichment of DEGs across the four floral developmental stages (S1–S4). Pathways are ranked from top to bottom according to *p*-value, with the most significant pathway at the top. Bubble color corresponds to *p*-value and bubble diameter corresponds to the count of enriched genes.

Next, KEGG enrichment analysis of DEGs identified two metabolic pathways significantly associated with floral scent during flower development: sesquiterpenoid and triterpenoid biosynthesis (ko00909) and phenylpropanoid biosynthesis (ko00940) (Figure 10).

**Figure 11:**
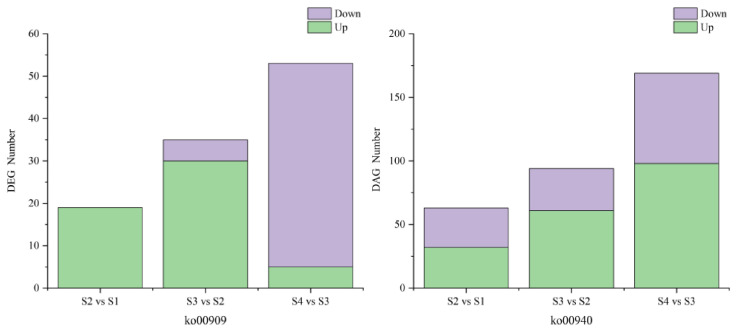
Number of DEGs in the ko00909 and ko00940 pathways in three pairwise developmental stage comparisons.

A comparison of the DEGs annotated in the two pathways showed that the gene expression trend of ko00909 was more consistent with class 1 of K-means cluster analyses (Figure 11), indicating that gene expression during floral growth is mainly related to VOC biosynthesis, specifically sesquiterpenoid and triterpenoid biosynthesis.

### 3.4. Integrative Analysis of Transcriptome and Metabolome

Differential VOCs and DEGs were subjected to integrative KEGG pathway enrichment analysis. The results showed that across the developmental stages (S1–S4), only one pathway related to floral scent formation was consistently enriched: the sesquiterpenoid and triterpenoid biosynthesis pathway (ko00909). Three VOCs (α-hemelene, δ-naphthalene, and β-bisabolene) and 15 DEGs were enriched in the sesquiterpenoid and triterpenoid biosynthesis pathway, exhibiting similar variation patterns across developmental stages. Among the 15 DEGs, nine with the highest expression levels may be involved in floral scent formation. The nine DEGs exhibited >91% homology to the alpha-humulene synthase (*ZSS1*) gene of *Dendrobium catenatum*, with five genes (cluster-42006.16, cluster-42006.23, cluster-42006.3, cluster-42006.30, and cluster-42006.8) showing 100% sequence coverage, indicating potential functional similarity to *ZSS1.*

An additional scent-associated pathway was that responsible for limonene and pinene degradation (ko00903), which was enriched during S2–S4. This pathway was enriched in two VOCs (α-pinene and l-perillyl alcohol) and three DEGs. Among these, two genes (cluster-58815.11 and cluster-40030.1) showed peak expression at the aging stage, which had 92.4% and 98.3% homology to aldehyde dehydrogenase family 3 member H1 (*ALDH*) of *D. catenatum*, with sequence coverage of 95% and 100%, respectively.

**Figure 12:**
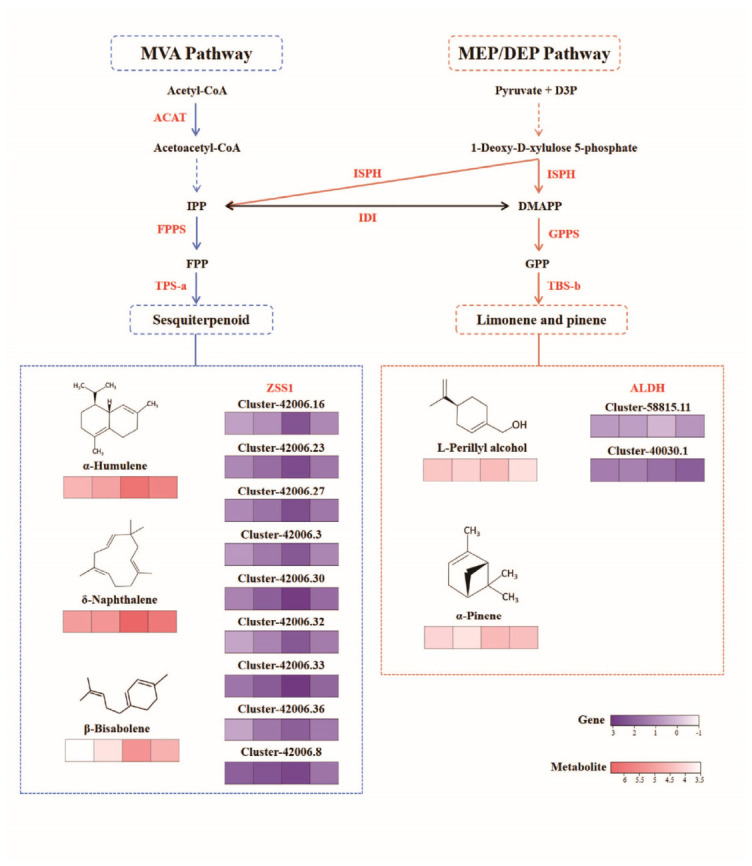
Terpenoid biosynthesis pathways associated with floral scent during flower development in *D. devonianum*. Colored bars represent the relative expression levels of genes or metabolites, with darker colors indicating higher levels. The heatmaps display standardized expression patterns of representative genes (purple gradient, scale: 3 to −1) and metabolites (red gradient, scale: 6 to 3.5). Each row corresponds to a specific gene cluster or metabolite, and the color intensity reflects the relative expression level across samples. Solid arrows denote enzymatic reactions catalyzed by labeled enzymes (e.g., ACAT, FPPS, TPS-a, ISPH, IDI, GPPS, TBS-b, ALDH). Dashed arrows represent intermediate metabolic steps or cross-pathway connections. Dashed boxes enclose the full pathway and its downstream terpenoid end-product branches.

**Figure 13:**
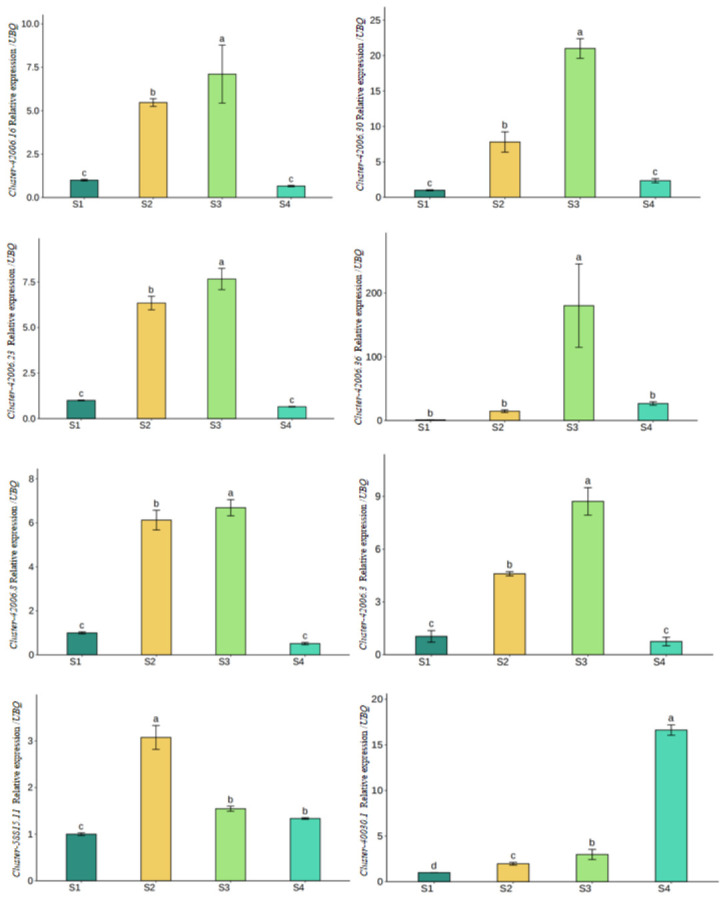
Quantitative reverse-transcription polymerase chain reaction (qRT-PCR) validation results. Different letters indicate significant differences among groups as determined by ANOVA followed by Tukey’s HSD post-hoc test (*p* < 0.05).

The pathway map of terpene aroma biosynthesis in *D. devonianum* flowers is shown in Figure 12, together with the changing trends of the eleven genes and associated metabolites. The eleven genes were further validated for qRT-PCR analysis, of which eight exhibited expression patterns consistent with the transcriptome data (Figure 13). The predicted coding sequences of three genes (cluster-42006.33, cluster-42006.32, and cluster-42006.27) could not be clearly resolved, likely due to their being transcript variants of the same gene.

ACAT, acyl coenzyme A-cholesterol acyltransferase; FPPS, farnesyl pyrophosphate synthase; TPS-a, sesquiterpene synthases of category a; TPS-b, sesquiterpene synthases of category b; ISPH, 4-hydroxy-3-methylbut-2-en-1-yl diphosphate reductase; GPPS, geranyl diphosphate synthase; ZSS1, alpha-humulene synthase; ALDH, aldehyde dehydrogenase (NAD+); IPP, isopentyl pyrophosphate isomerase; FPP, farnesyl pyrophosphate synthase; IDI, isopentenyl-diphosphate Delta-isomerase; G3P, glyceraldehyde 3-phosphate; DMAPP, dimethylallyl pyrophosphate; GPP, geranyl pyrophosphate ammonium salt; MVA, mevalonate pathway; MEP/DEP, methylerythritol phosphate pathway.

## 4. Discussion

### 4.1. Terpenoids, Esters, and Heterocyclic Compounds as Major Constituents of Floral Scent in D. devonianum

In this study, 664 VOCs were identified from *D. devonianum* flowers, with terpenoids, esters, and heterocyclic compounds as the dominant classes, most of which exhibited fruity and sweet characteristics. Among these, 2-isobutyl-3-ethoxypyrazine and hexanal showed the highest abundance across all developmental stages and did not vary significantly during flower development. However, their OAVs were below 1, indicating a limited contribution to floral scent.

VOCs exhibited dynamic changes during flower development, with the greatest accumulation observed at the blooming stage (S3), particularly for terpenoids. This pattern is consistent with previous studies in other *Dendrobium* species, where terpenoids were also identified as the major contributors to floral scent and peaked at the blooming stage, such as *Dendrobium chrysotoxum* Lindl., *Dendrobium moniliforme* (L.) Sw., and *Dendrobium loddigesii* Rolfe [23,24,25]. Based on an OAV threshold ≥ 1, five aroma-active VOCs were identified as major contributors to floral scent, including perillyl alcohol, L-perillyl alcohol, terpinen-4-ol, 2-(4-methylphenyl)propan-2-ol, and cis-3-hexenyl butyrate. Integrative analysis of metabolomic and transcriptomic data further identified five VOCs (α-humulene, β-bisabolene, δ-naphthalene, L-perillyl alcohol, and α-pinene) associated with enriched biosynthetic pathways. Taken together, nine VOCs were identified as putative key compounds contributing to the floral scent of *D. devonianum*.

Dendrobium flowers are rich in polysaccharides, flavonoids, and polyphenols, and exhibit strong antioxidant activity [26,27]; however, the nutritional and bioactive potential of their VOCs has been inadequately explored. In this study, most DEAVOCs from *D. devonianum* flowers were found to have strong aromas, and several have reported bioactivities, including treatment of respiratory infections, liver detoxification, nerve relaxation, and fatigue resistance [28,29,30]. For instance, α-hemelene and terpinen-4-ol, identified in this study, have demonstrated anti-inflammatory and antimicrobial effects in vitro [31,32], while perillyl alcohol and L-perillyl alcohol can induce apoptosis in tumor cells [33]. These findings suggest that *D. devonianum* flowers may hold potential as functional foods, either consumed directly or incorporated into beverages, meal replacement powders, or other products [34,35].

Floral VOCs also play important ecological roles, mediating interactions with pollinators and defending against herbivores and pathogens. The stage-specific VOC changes observed in *D. devonianum* indicate that emission may be developmentally regulated to optimize such ecological functions. For example, the increased accumulation of terpenoids and esters at the blooming stage (S3) could enhance floral attractiveness to pollinators by producing strong and characteristic scents. Conversely, changes in VOC profiles at later stages may be associated with defense-related functions, consistent with reports that certain terpenoids possess antimicrobial or anti-herbivore activity [12,36,37,38].

In addition, VOCs serve as natural antimicrobials and preservatives with antagonistic effects against insects and fungi [39,40]. Among the VOCs identified in this study, compounds such as pinene and cinnamic acid [41,42] can directly inhibit or kill pathogenic bacteria on vegetables and improve undesirable flavors in oxidized meat. These compounds may be applied in fast food and ready-to-eat dishes to enhance food safety, providing alternatives to chemical additives [43].

The accumulation of key VOCs may influence both the flavor and potential uses of flowers [44]. Different floral developmental stages affected the accumulation of volatile sesquiterpenes and esters, consistent with previous reports showing that sesquiterpenes largely determine the scent profiles of *D. catenatum* flowers, whereas monoterpene levels remain relatively stable throughout development [45,46]. The main VOCs of many aromatic Dendrobium species include β-stilbene, α-pinene, and limonene [24,46].

Our study also identified volatile esters that contribute substantially to floral scent. These low-molecular-weight compounds are highly expressed, display efficacies comparable to terpenes [47], and are generally safer for application [48,49]. Although esters have been primarily studied in the context of plant reproduction, their potential uses in food remain underexplored. Further investigations into the metabolic regulation of esters in relation to scent could facilitate the discovery and large-scale production of VOCs with desirable properties through artificial synthesis or pathway modulation.

### 4.2. Main Pathways of Scent Formation in D Devonianum Flowers

Based on integrative transcriptomic and metabolomic analyses, we constructed a terpene biosynthesis pathway underlying floral scent formation in *D. devonianum*. The dynamic changes in VOCs were primarily associated with sesquiterpenoid and triterpenoid biosynthesis, as well as limonene and pinene degradation. Sesquiterpenoid and triterpenoid biosynthesis occurs downstream of the MVA pathway, whereas limonene and pinene degradation is downstream of the MEP/DEX pathway [50,51]. Previous studies have shown that upstream and midstream enzymes in terpene metabolic pathways are generally highly conserved, with downstream enzymes susceptible to differential expression [20,52,53]. TPS-a and TPS-b are subfamilies of key genes that catalyze the formation of sesquiterpenes and monoterpenes, respectively [54,55,56]. Thus, the DEGs most relevant to aroma regulation in *D. devonianum* may be those encoding TPS-a and TPS-b. Monoterpenes are relatively stable, whereas monoterpenes such as limonene and pinene tend to degrade after the flowers reach a certain level of maturity [57]. Following blooming, flowers produce high levels of sesquiterpenes, both to attract pollinators and to defend against herbivorous insects or pathogenic microbes [58].

Eleven genes were identified as candidates involved in terpene metabolism, which were annotated as *ZSS1* and *ALDH* homologs. *ZSS1* encodes α-humulene synthase, a key enzyme catalyzing the formation of sesquiterpenes, which are important for floral scent production and may also contribute to plant defense and pollinator attraction [59]. Overexpression of *ALDH* genes in plants has been reported to induce abscisic acid accumulation and promote senescence [60]. Based on the combined expression patterns of DEGs and VOCs, α-pinene and L-perillyl alcohol are likely produced through these pathways, with *ALDH* genes potentially contributing to their downstream metabolism. qRT-PCR validation of the 11 candidate genes confirmed consistent expression trends for eight of them, suggesting these genes may be involved in regulating sesquiterpene biosynthesis and monoterpene metabolism in *D. devonianum* flowers. These results provide insights into the metabolic regulation of terpene biosynthesis and may guide future studies on transcriptional regulation of terpene synthases.

### 4.3. Limitations

Despite all the findings, our study has several limitations. First, the identification of VOCs was primarily based on spectral matching and retention indices without comprehensive confirmation using authentic standards. Therefore, the reported key VOCs should be considered as putative. Moreover, it should be noted that odor thresholds in water were used due to data availability, and these may differ from thresholds in air or natural floral matrices. Therefore, the calculated OAVs represent approximate estimates rather than absolute sensory contributions. Finally, the proposed pathways were based on integrative multi-omics analyses without experimental validation. Nevertheless, this approach provides a useful comparative framework for identifying potential key aroma compounds. Future studies should incorporate authentic standards and sensory-based analyses, as well as more accurate quantification approaches, to validate the identity and aroma contribution of these compounds across developmental stages.

## 5. Conclusions

This study examined differential VOCs throughout the development of *D. devonianum* flowers. To ensure optimal flavor when the flowers are used in food products, they should be collected during the blooming stage. With the current focus on healthy eating, *Dendrobium* flowers can provide raw material for the production of nutritious and functional foods.

## Figures and Tables

**Table 1:** Characteristic scent compounds of *Dendrobium devonianum*, with relative contents (GC–MS) and odor activity values (OAVs).

Compound	CAS	OAV *	Relative Content **				Odor
			S1 (log_10_)	S2 (log_10_)	S3 (log_10_)	S4 (log_10_)	
Perillyl alcohol	536-59-4	7.00	4.39	4.27	4.71	4.51	Green, cumin, spicy, aromatic, woody, cardamom, floral, waxy, violet
L-perillyl alcohol	18457-55-1	1.10	4.31	4.15	4.49	3.89	/
Terpinen-4-ol	562-74-3	1.20	4.23	4.50	5.44	5.78	Turpentine, nutmeg, musty
2-(4-Methylphenyl)propan-2-ol	1197-01-9	5.10	2.84	3.07	4.07	5.02	Sweet, fruity, cherry, coumarin, floral, camphor
Cis-3-hexenyl butyrate	16491-36-4	20.00	ND ***	ND ***	5.39	5.04	Fresh, green, apple, fruity, wine, metallic, buttery
2-Isobutyl-3-ethoxypyrazine	24683-00-9	0.01	7.58	7.62	7.62	7.55	Green bell pepper, pea, galbanum
Hexanal	66-25-1	0.01	7.30	7.33	7.25	7.13	Aldehyde, grassy, green, leafy, vinegar
α-humulene	6753-98-6	0.16	4.67	4.96	6.04	5.67	woody
δ-Naphthalene	483-76-1	0.01	5.08	5.22	6.36	5.99	Thyme, herbal, woody, dry
β-Bisabolene	495-61-4	/	3.52	3.88	5.28	4.71	balsamic, woody
α-Pinene	80-56-8	0.27	4.08	3.87	4.48	5.43	Fresh, camphor, sweet, pine, earthy, woody

* OAV: Odor Activity Value (based on relative contribution to overall odor). ** Relative content: log10-transformed peak area from GC–MS analysis. *** ND: not detected.

## Data Availability

The original contributions presented in the study are included in the article, further inquiries can be directed to the corresponding authors.

## Supplementary Materials

The following supporting information can be downloaded at: https://www.mdpi.com/article/10.3390/metabo16040276/s1. Table S1. Primer information. Table S2. Summary of RNA-seq sequencing statistics. Table S3. De novo assembly statistics for the RNA-seq data.

## Abbreviations

DEAVOCs	Aroma-conferring volatile organic compounds
VOCs	Volatile organic compounds
PCA	Principal component analysis
PLS-DA	Partial least-squares discriminant analysis
OAV	Odor activity value
qRT-PCR	Quantitative Reverse-transcription Polymerase Chain Reaction
DEG	Differentially expressed unigenes
DEM	Differentially expressed VOC metabolites
