# Supplementary material for: Unveiling the Fragrant Secrets of Dendrobium devonianum: Terpenoid Pathways and Floral Scent Dynamics
Source: Metabolites. 2026 Apr 18;16(4):276. doi: 10.3390/metabo16040276 (PMC13117177; doi:10.3390/metabo16040276)
Supplement: Supplementary file 1 [file metabolites-16-00276-s001.zip › metabolites-4162298-supplementary.pdf]

Supplementary files

Table S1. Primer information.

Gene Name		Sequence（5′-3′）	Product Size
Unigene 57087-PAL2	Forward	TGAGCGAGTACCGTAACCCC	165 bp
	Reverse	TCATGCTGTCCATAACCCAATC	
Unigene 74899-PAL	Forward	TCAATTCTCCGAGCTGGTCA	168 bp
	Reverse	TGGACGTGGTTGGTTACTGG	
Unigene 71603-C4H	Forward	CTGTCCTGGAATCATTCTGGC	161 bp
	Reverse	CAACAACAGTGGAGTGCTTCAATA	
Unigene 71608-C4H	Forward	CCTCTTAGCCCTCTTCTGTGC	154 bp
	Reverse	GTCGGTTAGGTTACGGTGGTT	
Unigene 84379-4CL5	Forward	AACGAATCACTCGATGTCACCA	121 bp
	Reverse	AATCAACCGCACGCCAGA	
Unigene 73540-4CL5	Forward	GTGGTGGATAGGTTGAAGGAGC	124 bp
	Reverse	CATCTGGAAACGGTATGACAGC	
Unigene 64592-CHS1	Forward	CCACAAGGCACATACTCAGCG	144 bp
	Reverse	CGAACCCAAACAGAACACCC	
Unigene 78988-CHS	Forward	GGACAACCCAAATCTAAGATAACC	115 bp
	Reverse	TCTTCACAGAGGGTCGGAGG	
Unigene 73709-F3H	Forward	AAGGGTGGCTTCATCGTCTC	170 bp
	Reverse	CCCATGAGGTTCTCGCTGTAT	
Unigene 29740-FLS	Forward	CAAGTGCCCACAGCCAGAA	168 bp
	Reverse	TGGTCAGCAAGCAAAACAGTAA	
Unigene 69590-FLS	Forward	ACATTGGTGACCAGCTTGAGAT	117 bp
	Reverse	CAGGTGGCGAGCAAAACA	
GAPDH	Forward	CTTCAACATCATCCCTAGCAGC	102 bp
	Reverse	GTGGGAACACGGAAGGACA	

Table S2. Summary of RNA-seq sequencing statistics.

Sample	Raw reads	Clean reads	Clean base (Gb)	Q20 (%)	Q30 (%)	GC content (%)
S1-1	47,810,628	45,737,908	6.86	97.82	93.59	47.13
S1-2	59,541,012	57,298,750	8.59	97.94	93.8	47.15
S1-3	51,477,160	49,736,120	7.46	97.87	93.67	47.04
S2-1	45,239,734	43,788,088	6.57	97.96	94.07	46.88
S2-2	50,642,500	49,115,462	7.37	97.86	93.67	47.37
S2-3	59,643,308	57,461,576	8.62	97.84	93.59	47.37
S3-1	43,470,842	42,005,594	6.30	97.79	93.50	46.92
S3-2	45,394,562	43,751,332	6.56	97.67	93.59	47.02
S3-3	47,902,742	45,718,976	6.86	97.44	92.91	46.53
S4-1	51,114,616	49,408,532	7.41	97.79	93.48	47.49
S4-2	46,694,750	45,124,218	6.77	97.7	93.34	47.35
S4-3	50,589,534	48,848,266	7.33	97.75	93.4	46.93

Table S3. De novo assembly statistics for the RNA-seq data.

Type	Number	Mean Length (bp)	N50 (bp)	N90 (bp)
Transcript	233,317	953	1640	367
Unigene	122,331	1263	1855	558
